# Health-care guidelines and policies during the COVID-19 pandemic in Mexico: A case of health-inequalities

**DOI:** 10.1016/j.hpopen.2020.100025

**Published:** 2020-12-15

**Authors:** Elysse Bautista-González, Jimena Werner-Sunderland, Paulina Pérez-Duarte Mendiola, Cesar Jeronimo Esquinca-Enríquez-de-la-Fuente, Daniela Bautista-Reyes, Maria Fernanda Maciel-Gutiérrez, Inkel Murguía-Arechiga, Cecilia Vindrola-Padros, Manuel Urbina-Fuentes

**Affiliations:** a Research Department of Epidemiology and Public Health, Institute of Epidemiology and Health-care, University College London, London, UK; b RREAL COVID-19 Policy Team, Mexico City, Mexico; c RREAL Director and Department of Targeted Intervention, Institute of Epidemiology and Health-care, University College London, London, UK; d Investigación en salud y demografía (INSAD), Mexico City, Mexico

**Keywords:** Health policy, Health inequalities, COVID-19, Pandemic, Universal health coverage, Mexico

## Abstract

**Background:**

Heterogeneous government responses have been reported in reaction to COVID-19. The aim of this study is to generate an exploratory review of healthcare policies published during COVID-19 by health-care institutions in Mexico. Analyzing policies within different health sub-systems becomes imperative in the Mexican case due to the longstanding fragmentation of the health-care system and health inequalities.

**Data and Methods:**

Policies purposely included in the analysis were published by four public health institutions (IMSS, ISSSTE, SSA and PEMEX) during the COVID-19 epidemic in Mexico (from February 29th to June 15th, 2020) on official institutional websites. Researchers reviewed each document and classified them into seven policy categories set by the Rapid Research Evaluation and Appraisal Lab (RREAL): public health response, health-care delivery, human resources, health-system infrastructure and supplies, clinical response, health-care management, and epidemiological surveillance.

**Results:**

Policy types varied by health institution. The largest number of policies were aimed at public health responses followed by health-care delivery and human resources. Policies were mainly published during the community transmission phase.

**Conclusions:**

The pandemic exposed underlying health-care system inequalities and a reactive rather than prepared response to the outbreak. Additionally, this study outlines potential policy gaps and delays in the response that could be avoided in the future.

## Highlights

• Ensure preparedness for new outbreaks through a proactive, preventive and less reactive system.
• Homogenize clinical and epidemiological surveillance processes across health institutions.
• Avoid disrupting preventive, clinical or palliative services through innovative solutions.
• Make PPE and psychological support available for health-care staff throughout the pandemic.
• Generate a single official platform to inform staff on updated clinical guidelines.

## 1 Background

Despite the international reporting, preparedness and collaboration mechanisms developed by the World Health Organization and other international actors, a variation in government responses to SARS-CoV-2 have been described around the world [1], [2], [3], [4]. Moreover, in the context of a pandemic, the health-care system fragmentation led to differences in the development of health guidelines and protocols [4], [5], [6], [7]. Therefore, analyzing the policies within the main health sub-systems across the different COVID-19 phases becomes imperative in Mexico.

The Mexican government defined three COVID-19 phases: confirmation of imported cases from abroad, confirmation of transversal transmission (phase two or mitigation phase) and confirmation of community transmission (phase three) [8]. These phases dictated the actions taken by the government in response to the pandemic at a national level [9].

The Mexican health-system is built from both private and public sectors. As a result, there are different sub-systems formulated according to the profile of users: the population that is privately insured, the publicly insured and the uninsured or open population [10], [11]. The largest public insurance institutions that embody the public health-system are: Instituto Mexicano del Seguro Social (IMSS), Instituto de Seguridad y Servicios Sociales de los Trabajadores del Estado (ISSSTE) and Petróleos Mexicanos (PEMEX) [11]. The largest institution that covers the uninsured population is the Secretaría de Salud (SSA) [11], [12]. The right to be covered by any of the other aforementioned institutions derives from formal employment within the institutions [12]. Thus, the affiliation to a specific sub-system will dictate the patient’s diagnosis, treatment and prognosis which will become embodied in the health-outcome of an individual [13], [14], [15]. A summary of the institutional differences is available in Appendix 1.

In this context, social inequalities are translated into healthcare inequalities. Therefore, the aim of this study is to generate an exploratory review [16] of healthcare policies published during the current COVID-19 pandemic, in order to shed light on the inequalities between health institutions, exemplified by their response during the COVID-19 epidemic in Mexico.

## 2 Data & methods

Using a rapid qualitative research methodology [17], data was collected by four members of the research team using purposive sampling of institutional policies accessible through official websites such as: *www.coronavirus.gob.mx**;* *www.gob.mx/salud**;* *www.gob.mx/issste**;* *www.imss.gob.mx*; *educacionensalud.imss.gob.mx**;* *site.inali.gob.mx**;* *dof.gob.mx**;* *www.pemex.com* *and* *coviduti.salud.gob.mx*. Each one of these members oversaw data emerging from a single institution every day. Policies included in the analysis was published from February 29th to June 15th, 2020. Data was extracted into a shared spreadsheet where it was subsequently organized by one member of the team into the conceptual framework developed by RREAL [18]. Lastly, the classification process using the conceptual framework was cross-checked by two members of the team. Policies were classified into seven categories (public health response, health-care delivery, human resources, health-system infrastructure and supplies, clinical response, health-care management and epidemiological surveillance) that could allow for the comparison of COVID-19 responses across countries under the same framework [18]. The framework became a dynamic working document that was modified as new policies emerged and were constantly added to the analysis. Furthermore, in order to further analyze the policies, these were classified according to: date of publication, the place where they were expected to be enforced, the implementers of the policy (i.e., the people who had to read and act on a particular task) and potential beneficiaries (i.e., policies ensuring PPE for staff directly benefits healthcare workers, whereas modifying the triage for COVID-19 patients directly benefits the health-system users).

## 3 Results

**Fig. 1:**
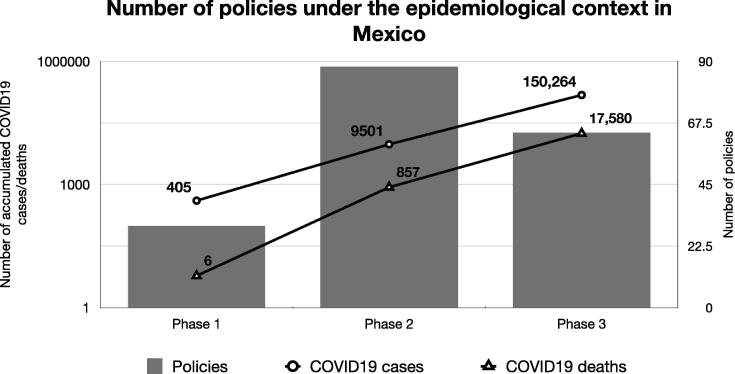
Overview of the number of policies published by COVID-19 phases under the Mexican epidemiological context (number of accumulated cases and deaths drawn on the Y axis). Phase 1 (February 29th to March 23rd); Phase 2 (March 24th to April 20th); & Phase 3 (April 21st to June 15th) in 2020.

From the four health institutions selected for the analysis, 182 national policies were identified. After stratifying by COVID-19 phases used by the Mexican government, results show 17% were published during phase one, 48% in phase two, and 35% in phase three (Fig. 1).

Thereafter, policies were classified by the REAL policy framework. As a result, the largest number of policies were aimed at public health response (25.3%), followed by health-care delivery (16.5%), human resources (15.4%), health-system infrastructure and supplies (14.8%), clinical response (13.7%), health-care management (9.9%) and epidemiological surveillance (4.4%); and fifty-nine different policy subcategories were identified. The definitions of each policy category and their subcategories are detailed in Appendix 2.

**Fig. 2:**
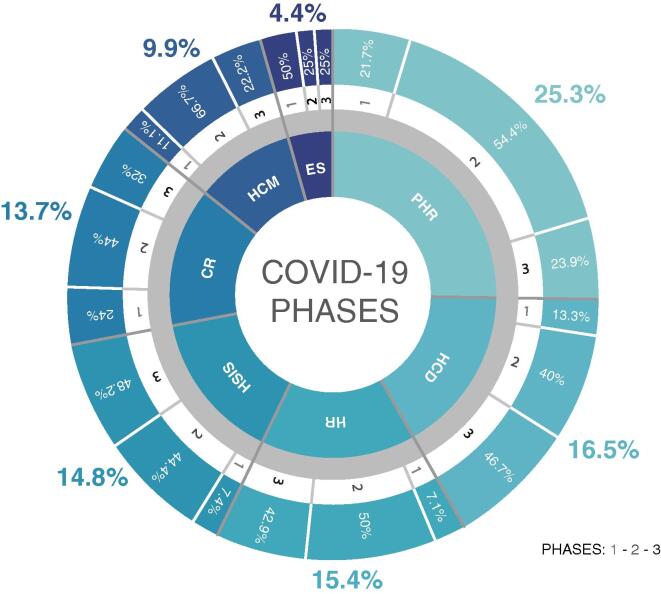
Type of policies published by four Mexican health-care institutions throughout the three COVID-19 phases using the RREAL policy category framework (PHR: Public health response, HCD: Health-care delivery, HR: Human resources, HSIS: Health-system infrastructure and supplies, CR: Clinical response, HCM: Health-care management and ES: Epidemiological surveillance).

Meanwhile public health response dominates the health-systems activities during the COVID-19 epidemic, results show policy categories were implemented at different stages (Fig. 2).

**Fig. 3:**
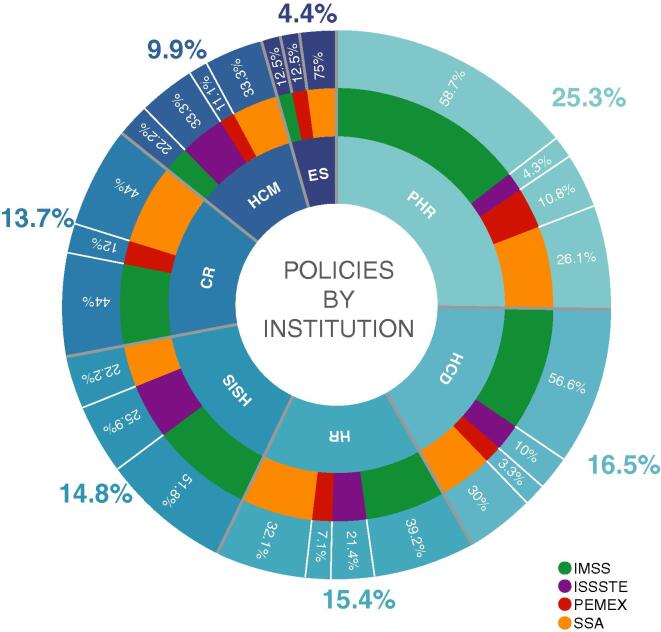
RREAL policy categories stratified by type of institution. The sum of the percentages by institution add up to 100% in each policy category. (PHR: Public health response, HCD: Health-care delivery, HR: Human resources, HSIS: Health-system infrastructure and supplies, CR: Clinical response, HCM: Health-care management and ES: Epidemiological surveillance).

**Fig. 4:**
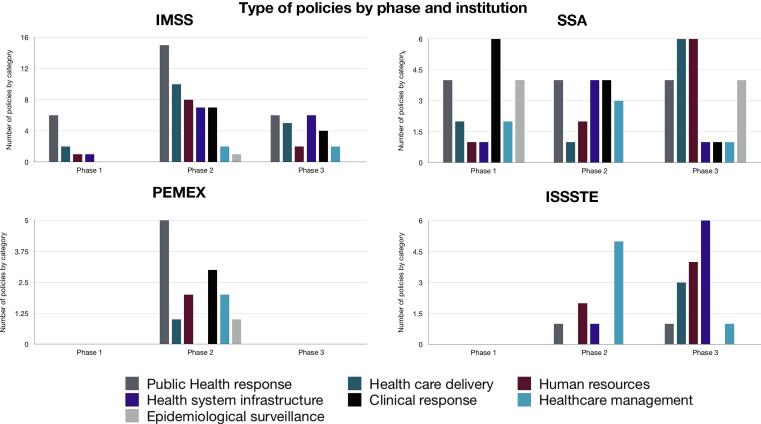
Comparison of the policies published by institutions throughout the COVID-19 phases in Mexico. Phase 1 (February 29th to March 23rd); Phase 2 (March 24th to April 20th); & Phase 3 (April 21st to June 15th) in 2020.

Overall, the institution that published the highest number of policies during the COVID-19 pandemic was the IMSS (46.7%), followed by the SSA (32.4%), the ISSSTE (13.2%) and PEMEX (7.7%). However, after stratifying each policy category by institution, health institutions show different levels of involvement in each policy category (Fig. 3). Furthermore, institutions not only published different policies, but they published policies at different times throughout the epidemic (Fig. 4). A summary of the policies by phase, location, implementers, beneficiaries and institution is available in Appendix 3.

Clinical response policies were published across all stages, particularly during phase 2. They sought to better the outcomes of COVID-19 patients within the hospital level. They were to be implemented by health-care professionals by building capacity and modifying processes, particularly for COVID-19 patients. This included COVID-19 nutritional, triage, screening, diagnosis and treatment algorithms. Meanwhile, some policies were continuously being updated on the different websites and uploaded with the same link, other documents were lost, and a new version was attached to the website, making the follow-up of updates impossible. Lastly, until phase three, the SSA’s developed more adapted content for the clinical response in indigenous communities. However, no adapted content for indigenous communities was published by any other health institution.

Epidemiological surveillance policies were implemented by health-care professionals and health-care providers and only benefited the COVID-19 population by establishing an algorithm for case confirmation, contact tracing and producing a death certificate. Epidemiological surveillance policies were implemented in hospital settings (75.0%), workplace (12.5%) and others (12.5%). The SSA developed three case confirmation algorithms in phase one and one in phase three. In contrast, the IMSS and PEMEX only generated a single document on case confirmation. However, these were not published until phase two. Only one algorithm for death certificates was published by the SSA, but not until phase 3.

Health-system infrastructure and supplies policies were implemented only in hospital environments by health-care professionals particularly during phases two and three. The population that mainly benefited from the expanding infrastructure (i.e., temporary hospitals, reconfiguration of hospitals, public private partnerships, shared hospital services), and supplies (i.e., PPE, ventilators, and sanitation products) were COVID-19 patients (77.8%), health-care professionals and the general population (through the re-conversion of breweries and maquila industries into alcohol gel and face masks-production factories) [19], [20].

Most of the health-care delivery policies were published in phase two and phase three. They were implemented by health-care professionals and health-care providers; and were directed to modify health-care delivery mechanisms for the general population (including housekeepers), vulnerable citizens (i.e., chronic disease patients, the elderly, oxygen dependent patients, pregnant women, and newborns) and COVID-19 patients. The policies employed changed the delivery of services in hospitals, at the workplace and generated health-care services in the community that could be accessible from home i.e., medical and mental health guidance, monitoring of all vulnerable patients, maternity or sick leaves were conducted remotely electronically or via phone. Additionally, family members were informed about the COVID-19 patient's status via phone. Moreover, the online payment of insurance fees was allowed early in the pandemic, as well as the refillable prescription for subsequent chronically ill patients. In contrast, the health-care delivery policies generated for hospitals included modifications in inpatient management and patient handover, particularly COVID-19 patients; whereas the rest of the vulnerable and general population’s delivery services were re-prioritized and experienced a reduction in the number of hospital visits.

Health-care management policies were implemented at the hospital level. However, in a few cases, it included guidelines for corpse control in funeral homes, aimed at controlling and preventing infections i.e., in petrol platforms. They were implemented by health-care professionals and benefited the general population, COVID-19 patients, vulnerable citizens (including pregnant women and newborns) and health-care professionals themselves. The health-care management policies aimed to benefit the COVID-19 patients through the approval of screening tests and the creation of a situation room that visualized the number of cases and beds. The policies published also served the general community through the deployment of the national guard to secure hospitals (phase 2), corpse control and infection prevention guidelines. Lastly, management policies that benefited health professionals were focused on guidelines for personal protective equipment (phase 2), managing the response team and preventing infection at the workplace.

Human resource policies were implemented at the hospital level and focused mainly on a broader range of health-care professionals rather than particular health-care providers. Phase one included capacity building and suspension of activities for non-essential workers and health-care professionals with risk factors. In phase two, 50% of the policies for human resources were published and included building capacity on COVID-19 related topics and managing discrimination against staff. Lastly, in phase three, human resource policies included: capacity building, bringing in additional human resources, space shifts, re-integration of medical students to hospital, and assigning a specific member of staff responsible for delivering distressful information to the patient’s family. Policies oriented at to benefitting health professionals included ensuring a proper resting space, having a residential complex particularly for clinicians, and providing mental health services for staff members. Economic stimuli for COVID-19 first responders were not published until phase three.

Public-health response policies are the most common type of policy used throughout the pandemic, across institutions and phases. Most public health policies were developed in phase two and three. During phase one, the policies developed included disease prevention, health promotion, social distancing, and stay at-home campaigns. During phase two, self-isolation for travelers was added. Lastly, but not until phase three, mental health campaigns were developed in order to avoid depression within the elderly and to aid the general population in coping with the mental health effects of the pandemic. These policies aimed to benefit the general population (including children and adolescents) and in lower prevalence the vulnerable patients (i.e., chronic disease patients, diabetics, disabled, elderly, heart disease, hypertensive, indigenous communities, inmates, obese, pregnant women, newborns and transplant patients).

Policies were stratified by place. They were mainly acting in hospitals (63.7%), community (28.0%), workplace (4.4%), child-care centers (0.6%), and other (3.3%) i.e., shelters, nursing homes, psychiatric hospitals, psychosocial rehabilitation centers, prisons and funeral homes.

## 4 Discussion

The first COVID-19 cases were reported in February 2020 [9]. Technical guidance documents on improving capacity to detect, prepare and respond to the outbreak have been published by the WHO since January 23 [21], [22] and the Mexican government developed its response strategy almost two months after the SARS-CoV-2 outbreak was reported by the Chinese government, and one month after the Emergency Committee (convened by the WHO) determined COVID-19 a public health emergency of international concern. As a result, the Mexican government had several weeks to deploy a response and preparedness plan, in liaison with the international public health agency of the United Nations and its national technical interlocutor, the SSA. However, the limitations for cooperation at the science–policy–society interface found in the global health-system and the international medical scientific community seemed to echo in the Mexican health-system during the COVID-19 pandemic [3].

After the first General Health Council (Consejo de Salubridad General, CSG) emergency meeting on March 19th, 2020, COVID-19 was recognized as a serious epidemic of primary level importance in Mexico. In case of health emergencies, the CSG is the national government entity chaired by the SSA (with the same level of authority as the president), legally enabled to emit, implement and enforce the observance of norms in Mexico. However, several irregularities affected the operation of the CSG, adding to the heterogeneous response. Besides the late timing of both the meeting and declaration, the CSG undermined its regular legal capacities by stating that prevention and control measures for COVID-19 would be established in consensus with other federal government institutions and state authorities [23]. This added further disruption to the response by politicizing every action mandated by the CSG [24]. Results from this study show the international and national dissonance was present within and between the different health-care providers’ policies, guidelines and recommendations.

Case confirmation algorithms were continuously changed throughout the pandemic and varied by institution. In addition, access to diagnostic resources varied across institutions and clinical settings. Thus, the lack of consistency and delay on the case confirmation process and specific guidelines to fill death-certificates might have led to negative effects on epidemiological surveillance of COVID-19 throughout the pandemic.

The limited capacity for local production, translation and adaptation of scientific evidence [25], in addition to the demand for COVID-19 information, interventions and policies, resulted in an important delay in the healthcare system response during the first months of the pandemic [26], [27]. In fact, some of the key guidelines needed for a clinical response were not provided until phase 3. Another setback to the clinical response, was the frequent adjustment, contradiction and updates on clinical diagnostic criteria and overall treatment provided. In addition, the lack of continuity of the virtual location of previous documents available (links) and lack of user-friendly platforms hindered the health-professionals' ability to access valuable information. Thus, clinicians were challenged to search, manage and appraise an unprecedented amount of scientific evidence during the COVID-19 response [28].

Only 7.4% of infrastructure and supplies policies were published in phase one. The expansion of the hospital infrastructure and the new public private partnerships came once the number of cases had started to increase significantly. Furthermore, the medical supply shortage prompted the reconversion of industries, and massive purchases from other countries with questionable quality standards [19], [29], [30]. Hence, the health sector was not adequately prepared to respond to COVID-19.

Moreover, the lack of mechanical ventilation and intensive care support infrastructure and supplies shed light on the deficiency of the supply chains and distribution process. In fact, Mexico had previously recognized these supplies as playing a central role in the swine-flu outcomes in the 2009 national epidemic [31], [32]. However, adequate preparedness strategies in relation to ventilators and the supply of Personal Protective Equipment (PPE) remains overdue.

The development of algorithms for the disposal of corpse control was chaotic, fragmented and late. It created confusion among the hospitals about how to dispose of an ever-increasing number of corpses. This led to conflicting numbers between the data shown by the Ministry of Health and the Civil Record Office [33], [34] and prompted some individual states to develop their own legal framework and guidelines on how to manage corpses and correctly codify the cause of death [35]. Additionally, it also fueled social discontent caused by the impossibility of families to mourn the death of their relatives following their general practices, customs and usages dictated [36]. Governance in this regard was central to avoiding social and political unrest.

Human resource policies were focused on building capacity and ensuring the health sector had sufficient staff and appropriate and sufficient resources available in the workplace. Although suspension of activities for non-essential workers, economic stimuli for incoming staff, spacing shifts and vacation days were used in some instances, human resources employed in the response to COVID-19 in many institutions were not enough to cover the deficit left with the withdrawal of pre-med interns and high-risk individuals [37], [38]. In the future, the allocation of a national budget for health-care personnel should be sustained if not increased to avoid the lack of qualified personnel in place during the response to a crisis. In addition, there should be a shift in academic training after COVID-19, for example, introducing new training in skills such as telemedicine or preparing students and residents to mitigate epidemics through more innovative and crisis-oriented educational approaches [39].

As a result of the work burden placed on the limited human resources in the health system, physical and psychological manifestations of stress arose in large numbers of health-care professionals [40], [41], [42]. Thus, psychological containment available for the health workers and the general population should not be delayed and should be a priority during a pandemic. Lastly, the safety of the health personnel should be considered not only with the insurance of PPE [43], but also, with the capacity building and dissemination of how to deal with discrimination, stigmatization, and violence against them [44].

The epidemic modified health-care delivery across all institutions for both COVID-19 and non-COVID-19 populations. These modifications represented 16.5% of the total amount of policies published during the epidemic, making it the second most common policy during the COVID-19 epidemic in Mexico. Unfortunately, actions aimed at modifying the health-care delivery system were implemented during the community transmission phase. In consequence, a potential reduction in the transmission rates could have been achieved if the different health institutions were prepared to deliver health in a more risk averse fashion. Furthermore, modifications to the health-care system expedited potential changes that were not accounted for in the health-system’s annual objectives or budgets i.e., increasing digital health solutions. In the near future, potential policies to increase digital capacities i.e., telemedicine will strengthen the health-system’s digital health-care delivery preparedness.

Pandemics usually generate a shift and re-prioritization in public health responses [45]. This pandemic shifted the focus to the most pressing matter: COVID-19. Nonetheless, the country was simultaneously facing other epidemics i.e., obesity, diabetes, measles and violence [46], [47], [48], [49], [50], [51], [52], [53], [54], [55]. However, despite national and international concerns on these topics, no nutritional or exercise alternatives were developed to aid the population throughout the stay-at-home campaigns; immunization activities became disrupted [55], [56]; and no programs have been developed to address gender violence, injuries and addictions during the COVID-19 epidemic in Mexico.

Public health campaigns were merely “recommendations” issued at the national level that suggested social distancing or staying at home. These were confusing for many, as staying at home and then being told to only socially-distance yourself created a sense of uncertainty [57]. Additionally, compliance with stay-at-home or social-distancing guidelines became divergent between political parties and the local and federal governments.

Both stay-at-home or social-distancing guidelines neglect the fact that most people work in the informal sector in Mexico (i.e., as merchants, housekeepers) and are not able to be socially distant and avoid staying at home. Nonetheless, because strict compliance with recommendations was never targeted as an objective, only the population with the capacity to work from home were able to stick to the guidelines. Therefore, social inequalities furthered the health inequalities experienced by the Mexican population during the COVID-19 pandemic.

The content published for overcrowded places with a high risk of transmission was scarce. In addition, whilst all institutions established workplace policies, the SSA missed the opportunity to establish policies in the informal sector’s workplace. In Mexico, almost 60% of the population works in the informal sector [58], [59], [60]. As a result, this led to several outbreaks in crowded and busy places like markets and the subway [61], [62]. Thus, preparedness policies rather than reactive policies should be put in place to ensure physical distancing and adequate water and sanitation in over-crowded spaces including markets, shelters, transportation systems etc. This experience can potentially lead to policy modifications on how cities are built.

From the total policies found, most were meant to be implemented by health-care professionals and health-care providers (clinicians). But less responsibility is granted to the general public. In contrast, the population that benefits from the policies were COVID-19 patients, the general population, but less so health-care professionals, clinicians and vulnerable citizens. Thus, health-professionals carry an unjust and large burden, but policies rarely benefit them directly. Additionally, this sheds light on the fact that vulnerable populations (i.e., chronically ill, elderly, migrant or indigenous people) tend to suffer the most during an epidemic [63], due to the massive neglect of policies oriented to target this population and their needs.

Although the assessment of context and the policy gaps around the COVID-19 response has been achieved through this rapid policy review, time constraints prompted limited access to other data sources and might have limited the data collection process. This limitation has been widely described in the literature [17]. Moreover, this study only takes into account policies published from February to June 2020. We acknowledge that new policies were published after we finished our study. Hence, the policies reviewed for this study do not fully represent all guides, activities and modifications in the health sector during the COVID-19 outbreak in Mexico. The results cannot be considered as those produced by a systematic policy review, but rather as a snapshot of the policies accessible to the public in a specific period of time. Other policies outside the health sector were not taken into account.

## 5 Conclusions

The pandemic exposed underlying health-care system deficiencies, inequalities and lack of preparedness for the response to the outbreak. Health-care institutions not only prompted heterogeneous responses that potentially generated more inequalities among the population, but the nature of the response duplicated efforts that could have been conducted homogeneously through a single effort at an even earlier stage. Thus, a key lesson from the impact of the COVID-19 pandemic in Mexico is the value of health-care system unification and effective governance at the state and federal level for a more efficient preparation and early response. This requires the role of the General Health Council to be amplified and respected.

Preparing for an outbreak requires collaborating with potentially new political stakeholders and institutions. Nevertheless, it is evident that an intersectoral and inter-state governance and collaboration should be readily available for a crisis. Understanding the universe of stakeholders (both implementers and beneficiaries), sheds light not only on the policy gaps but also on the potentially relevant actors to include in the discussion for emergency preparedness and response.

There is an inherent need to improve the health information systems in Mexico. Not only to collect more reliable and timely epidemiological data to inform changes in the response, but also to provide access to updated scientific information in a more efficient manner. This has the potential to enhance the overall clinical response. In addition, the use of universal guidelines and policies across all institutions, might simplify the job of those working in the front-line providing care.

Technology should be integrated into health institutions in order to support the safe, effective and efficient delivery of services for all the population. This includes developing appropriate delivery services for vulnerable populations (i.e., disabled, indigenous and population not able to read or write) beyond the COVID-19 epidemic.

During the COVID-19 pandemic in Mexico, efforts were made to address previously neglected subjects like mental health or improve the coding of death certificates and publishing information into other languages and dialects. However, these efforts need to be integrated and maintained beyond the epidemic from the tertiary level all the way down to primary care settings, together with community participation strategies.

Lastly, stakeholders should aim for the unification of the health system in order to avoid further health outcome inequalities during and beyond the COVID-19 pandemic. More research needs to be done to understand if health inequalities and the social determinants of health have widened between the different institutions' populations. We expect this study to lead other countries’ policy comparisons in response to COVID-19.

## 6 Source(s) of support/funding

University College London contributed towards the open access publication fee. Fundación Mexicana para la salud (FUNSALUD) supported the preparation of this publication.

## 7 Disclosure of relationships and activities (ie, conflict of interests)

Jimena Werner-Sunderland certifies that she has had no privilege on the acquisition of information, despite having work affiliation with the Institute of Public Health of the State of Guanajuato (ISAPEG), which is part of the Ministry of Health (SSA). Both the Elysse Bautista-Gonzalez and Jimena Werner work as independent consultants in FUNSALUD.

## 8 Ethical issues

There was no need for the ethics committee to approve this research, as the policies included in the policy review are publicly available.

## CRediT authorship contribution statement

**Elysse Bautista-González:** Conceptualization, Methodology, Formal analysis, Investigation, Writing - original draft, Visualization, Supervision, Project administration, Funding acquisition. **Jimena Werner-Sunderland:** Investigation, Formal analysis, Writing - review & editing. **Paulina Pérez-Duarte Mendiola:** Investigation, Writing - review & editing. **Cesar Jeronimo Esquinca-Enríquez-de-la-Fuente:** Formal analysis, Writing - review & editing. **Daniela Bautista-Reyes:** Investigation, Investigation, Writing - review & editing. **Maria Fernanda Maciel-Gutiérrez:** Investigation, Visualization. **Inkel Murguía:** Investigation. **Cecilia Vindrola-Padros:** Writing - review & editing, Supervision. **Manuel Urbina-Fuentes:** Writing - review & editing, Supervision.

## Declaration of Competing Interest

The authors declare that they have no known competing financial interests or personal relationships that could have appeared to influence the work reported in this paper.

## Figures and Tables

**Table:** 

**Health indicators**	**PEMEX**	**SSA**	**IMSS**	**ISSSTE**
Year of creation	1938	1943	1944	1960
Type of affiliates	Public insurance (petroleum company)	Uninsured (open population)	Public insurance (private companies)	Public insurance (government workers)
Number of affiliates	12 million people	55 million	62 million	13 million
Annual budget spent per/capita in MXN	$8761	$2852	$3725	$4031
Doctors per 1000	7.2	1.8	1.7	3
Nurses per 1000	7.5	2.6	2.3	3.1
Beds per 1000	3.7	1.2	1.1	1.6

**Table:** 

***RREAL policy category***	***Definition***	***Sub-Category***
**Clinical response n = 25**	COVID-19 guidelines or algorithm to screen, triage, diagnose and treat a patient	Covid care algorithm
		COVID Triage
		Diagnosis and Screening
		Diagnosis and treatment
		Treatment and drug interactions
**Epidemiological surveillance n = 8**	Guidelines or algorithms to trace contacts, confirm cases and generate a COVID19 death certificate	Case confirmation algorithm
		Contact tracing algorithm
		Death certificate algorithm
**Health system infrastructure and supplies n = 27**	Policies looking to expand the public sector’s infrastructure through public–private partnerships, acquisition of supplies and donations	Additional sanitation resources
		Additional ventilators
		Ambulances redistribution
		Hospital reconfiguration
		Personal Protective equipment
		Resource Allocation
		Shared hospital infrastructure between institutions
		Temporary hospitals
**Health-care delivery n = 30**	Policies seeking to change the delivery of care in hospitals for COVID19 patients and family members; and change the delivery of health services for non-covid-19 patients	COVID Sick leave algorithm
		Informing death of family members
		Inpatient management
		Integrated patient care
		Maternity leave (online)
		Medical guidance (via phone)
		Mental Health services
		Online payment of fees
		Patient handover
		Prioritization of care
		re-prioritization of surgeries
		Reduction in hospital visits
		Refillable prescription
		Remote monitoring of patients
		Sick leave (online application)
		Telephone report on patient's status
**Health-care management n = 18**	Policies looking to effectively manage resources (i.e., PPE, hospital beds, screening tests), ensure hygiene and sanitation in workspace	Approval of screening tests
		Corpse control
		Deployment of national guard
		Infection Prevention and Control
		Personal Protective equipment
		Response team management
		Sanitation and cleaning of facilities
		Situation Room COVID-19
**Human resources n = 28**	Policies looking to manage human resources and their needs across the distinct stages of the pandemic, build capacity, generate economic stimuli among health-care staff and promote their mental health.	Additional Human resources
		Appropiate resting space
		Capacity bulding
		Covid capacity building
		Designate person to manage bad news
		Economic stimuli for COVID staff
		Economic stimuli for incoming staff
		Managing discrimination against staff
		Mental Health services
		Postpone vacation periods, days off, and or leave of absence
		Re-integration of students to medical units
		Residencial complex for clinicians
		Spacing shifts
		Stay-at-home campaign
		Suspension of activities for Non-essential workers
**Public health response n = 46**	Policies seeking to promote physical and mental health, prevent COVID-19 infection and disease spread and change the way people should distance themselves from others across the community.	“New Normal protocol”
		Disease prevention campaigns
		Health promotion campaigns
		Mental Health campaign
		Self-isolation
		Social distancing
		Stay-at-home campaign

**Table:** 

**Policy category**	**Policy category implementers**	**Policy category beneficiaries**	**Policy category prevalence by institution**
**Public health response**	General public 50%	General Population 65.22%	IMSS 58.7%
	Health-care Professionals 32.61%		ISSSTE 4.35%
	Vulnerable patients 15.22%	Vulnerable citizens 34.78%	PEMEX 10.87%
	People who travelled 2.17%		SSA 26.08%
**Health-care delivery**	Health-care professionals 83.33%	COVID-19 Patients 33.33%	IMSS 56.67%
		General Population 26.67%	ISSSTE 10%
	Health-care providers 16.67%	Vulnerable citizens 40%	PEMEX 3.33%
			SSA 30%
**Human resources**	Health-care professionals 89.29%	General Population 3.57%	IMSS 39.29%
		Health-care professionals 67.86%	ISSSTE 21.43%
	Health-care providers 10.71%	Health-care providers 21.43%	PEMEX 7.14%
		Vulnerable citizens 7.14%	SSA 32.14%
**Health-system infrastructure and supplies**	Health-care professionals 100%	COVID-19 Patients 77.78%	IMSS 51.85%
		General Population 3.70%	ISSSTE 25.93%
		Health-care professionals 18.52%	SSA 22.22%
**Clinical response**	General public 8%	COVID-19 Patients 72%	IMSS 44%
	Health-care professionals 40%	General Population 16%	PEMEX 12%
	Health-care providers 52%	Vulnerable citizens 12%	SSA 44%
**Health-care management**	Health-care professionals 100%	COVID19 Patients 11.11%	IMSS 22.22%
		General Population 55.56%	ISSSTE 33.33%
		Health-care professionals 27.78%	PEMEX 11.11%
		Vulnerable citizens 5.55%	SSA 33.33
**Epidemiological surveillance**	Health-care professionals 87.5%	COVID19 Patients 100%	IMSS 12.5%
			PEMEX 12.5%
	Health-care providers 12.5%		SSA 75%

**Figure:**
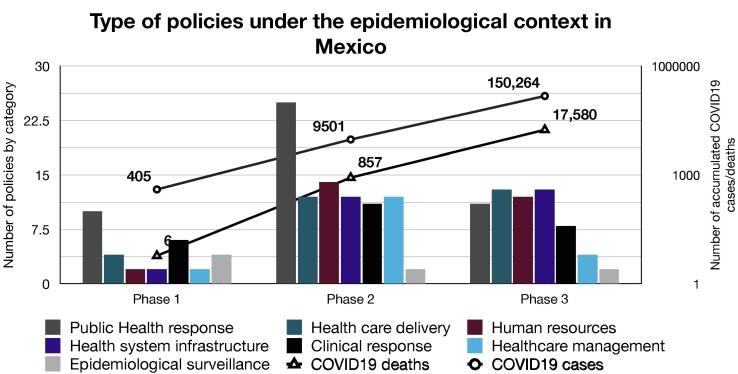


## Appendix A Health indicators from the four main health sub-systems in Mexico

An overview of the differences between the four health-care institutions selected for this study selected for this study. This table excludes the private sector and does not consider duplication of coverage. The annual budget spent per capita is in Mexican pesos. Health indicators were drawn from a comparative analysis developed by the Centro de Investigaciones Económicas y Presupuestarias (CIEP) [12].
